# Supplementary material for: Distribution and Implications of Haloarchaeal Plasmids Disseminated in Self-Encoded Plasmid Vesicles
Source: Microorganisms. 2023 Dec 19;12(1):5. doi: 10.3390/microorganisms12010005 (PMC10818511; doi:10.3390/microorganisms12010005)
Supplement: Supplementary file 1 [file microorganisms-12-00005-s001.zip › Supplementary Files/Supplementary Material.pdf]

Supplementary Material

Supplementary Material **1**
Figure S1: Overview of HMM profile generation for each ORF. 1
Figure S2: Flexible region of apHPVs. 2
Figure S3: Expanded view of core protein clusters. 3
Figure S4: Foldseek alignments of selected core-proteins of apHPVs. 4
Figure S5: Predicted structure of ORF6 of pR1SE 5
Figure S6: Extended arrays of antiparallel beta sheets in ORF6 6
Figure S7: C-Terminus of four selected ORF6 homologs 7
Supplementary Tables 7

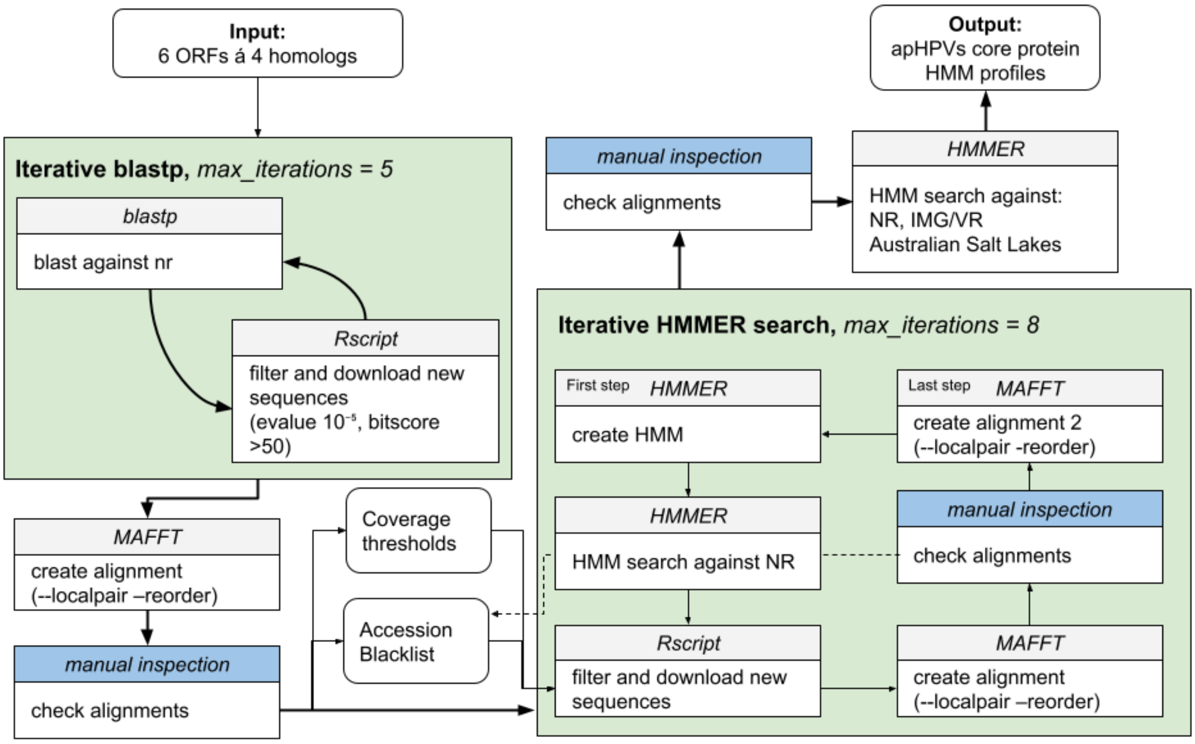

Figure S1: Overview of HMM profile generation for each ORF.

Details are given in method section 2.2. In brief, each ORF and homologs were iteratively blasted against NR, an alignment was created and curated. Then an iterative HMM search against NR and Australian Salt Lake proteomes was performed, inflating the HMM profiles. Each alignment was manually inspected before the next iteration. Sequences that were removed in one of the inspections, were added to an 'accession blacklist', which excluded them from being added again at a later iteration.

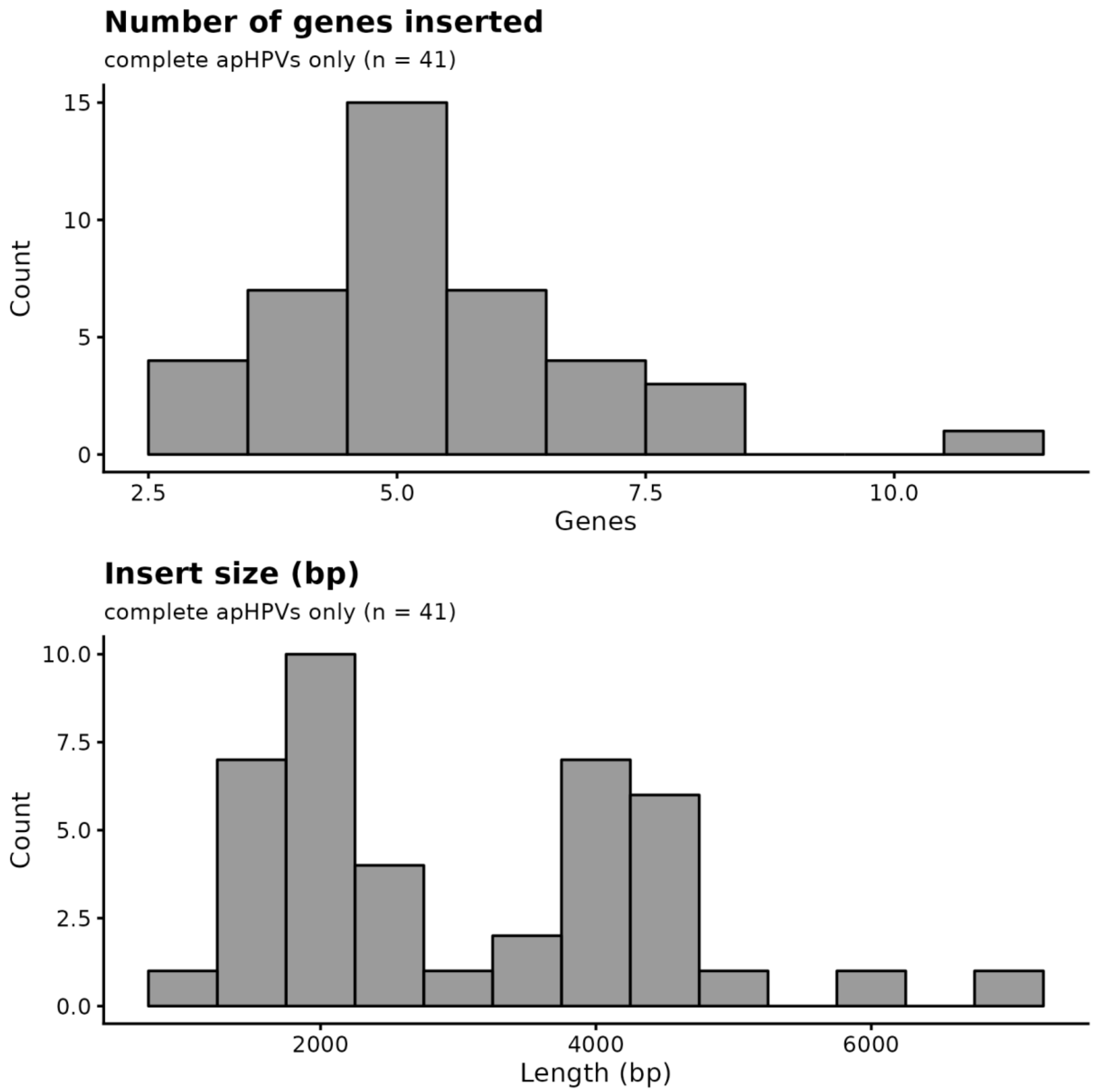

Figure S2: Flexible region of apHPVs.

Number of inserted genes and size (bucket size 500 bp) of the insert between cluster 1 (ORF6-9) and cluster 2 (ORF17-25) of 41 apHPVs.

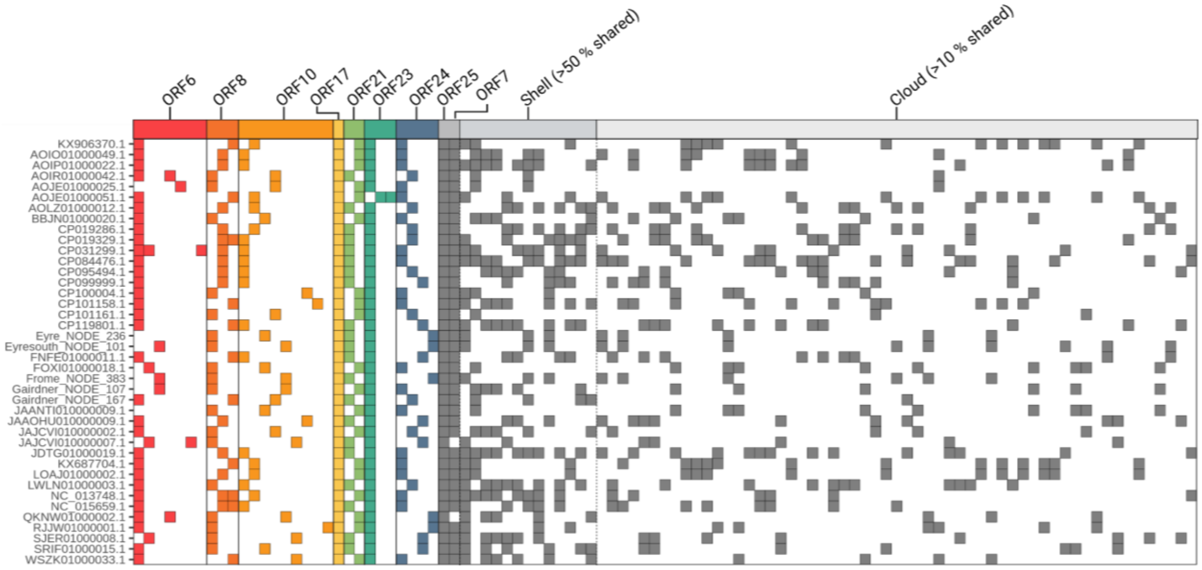

Figure S3: Expanded view of core protein clusters.

Presence-absence plot of protein clusters (columns) in different apHPVs (rows), in contrast to Figure 1 in the manuscript, the protein clusters of ORF6, 8, 10, 21, 23 and 24 are expanded.

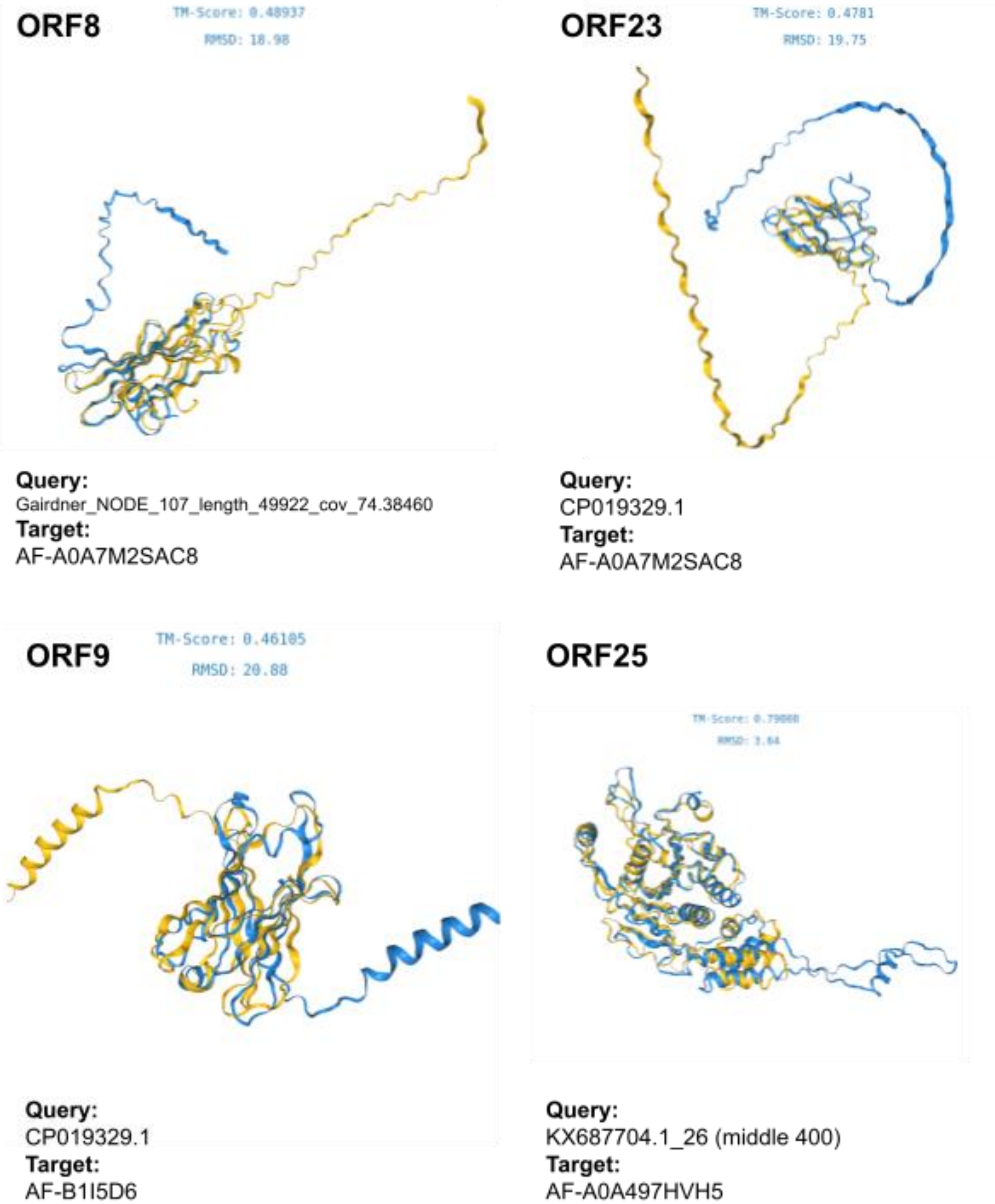

Figure S4: Foldseek alignments of selected core-proteins of apHPVs.

Selected examples of foldseek alignments of core proteins (blue) with publicly available protein structures (yellow). RMSD values and TM-scores are indicated on top. Detailed search results are documented in Table S2.

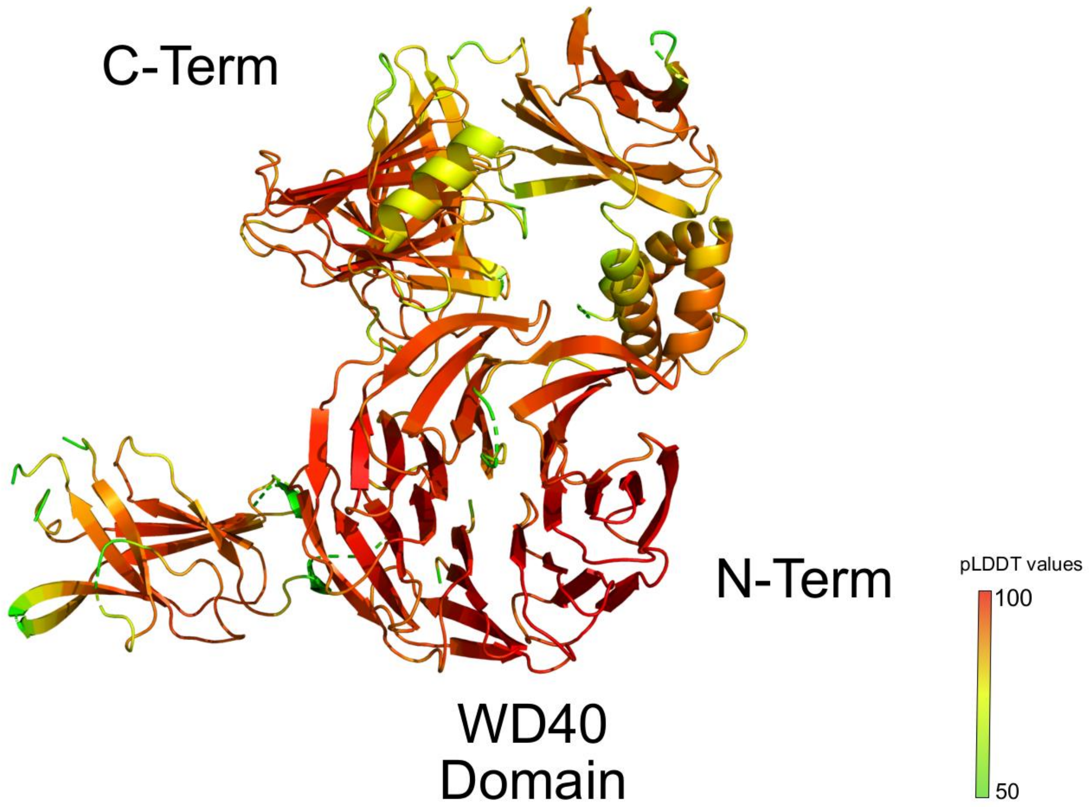

Figure S5: Predicted structure of ORF6 of pR1SE

Visible is a WD40 domain (IPR001680) at the N-terminus, found in ~56 % of all ORF6 homologs. Prediction was done using AlphaFold, color indicates pLDDT values.

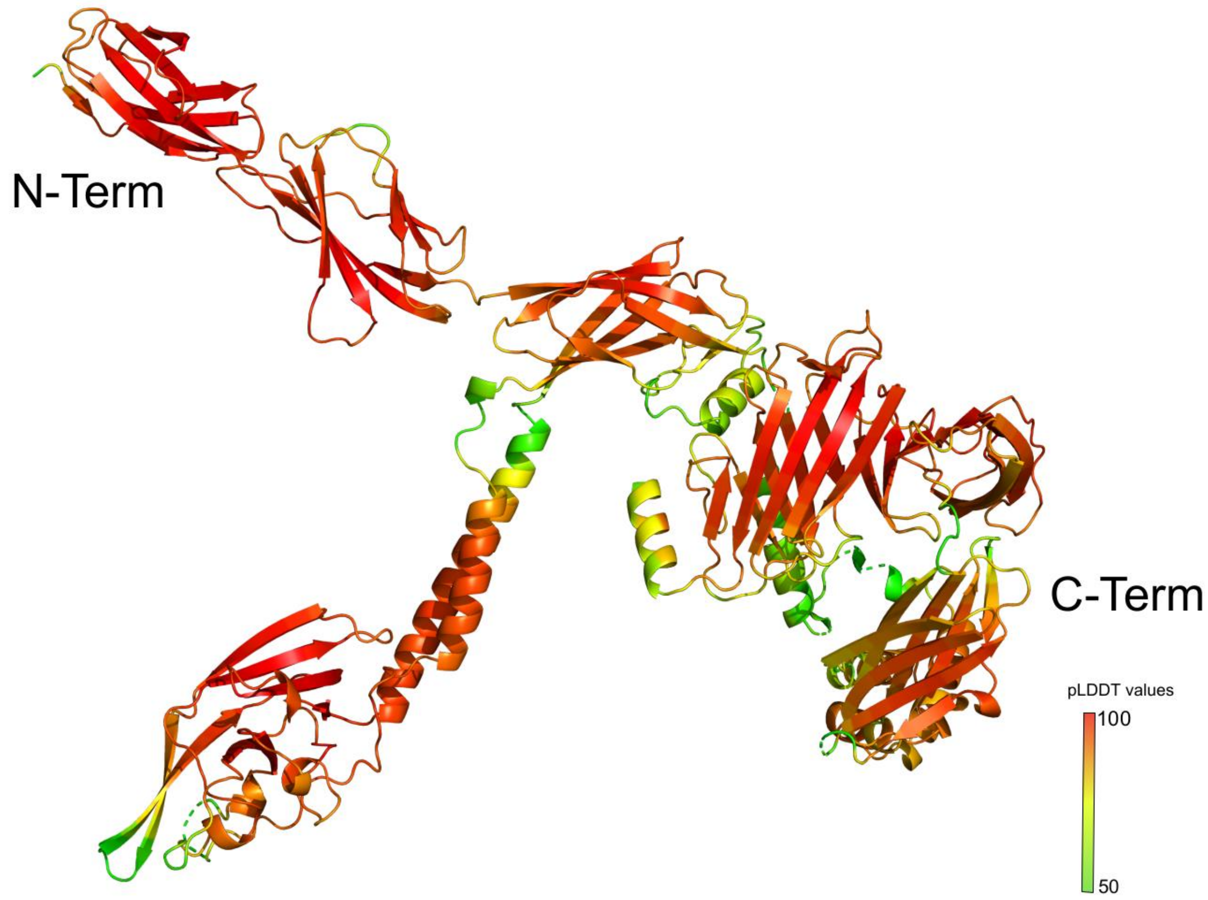

Figure S6: Extended arrays of antiparallel beta sheets in ORF6

Visible is a WD40 domain (IPR001680) at the N-terminus of the ORF6 homolog of apHPV BBJN01000020.1. Prediction was done using AlphaFold, color indicates pLDDT values.

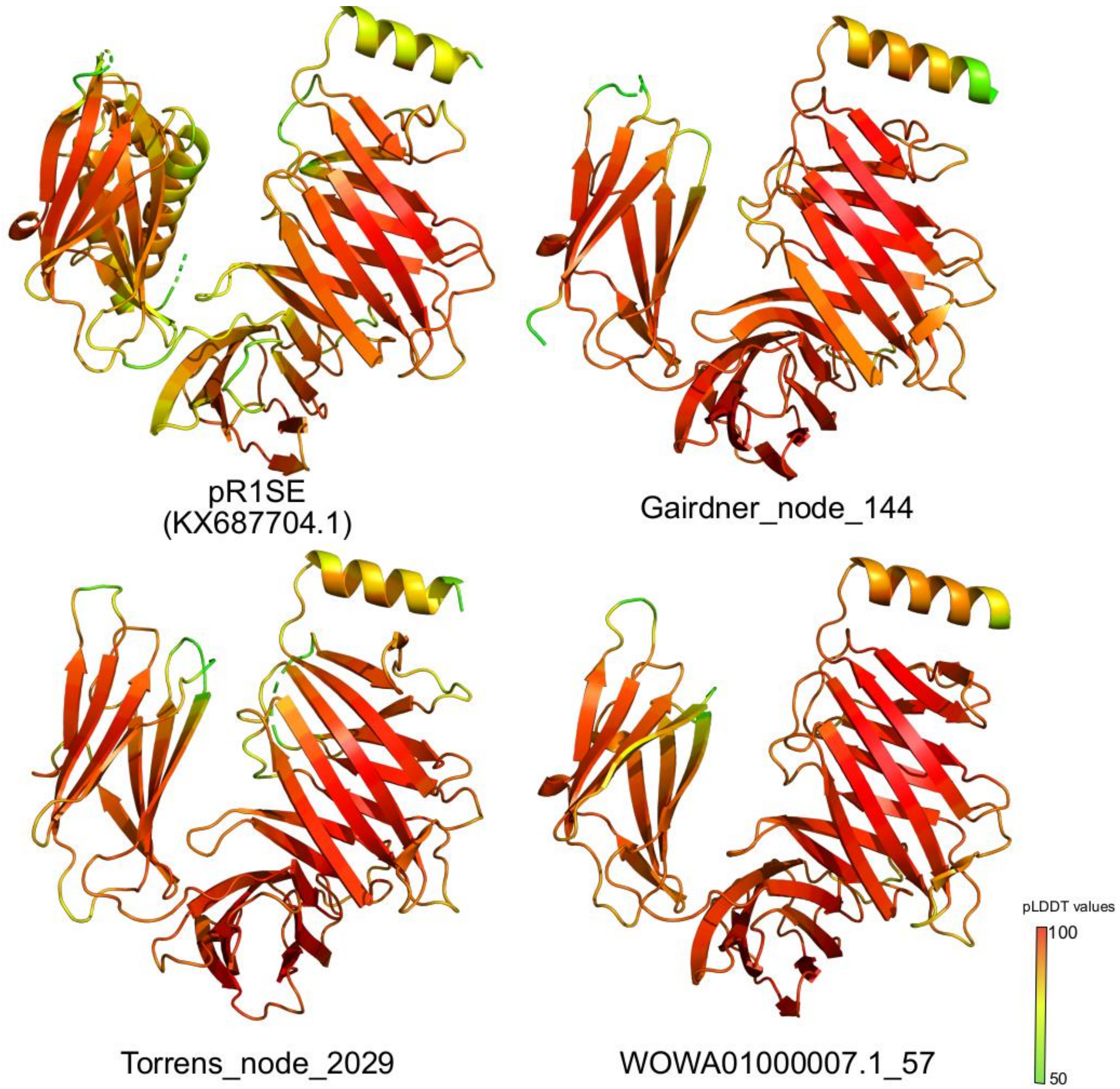

Figure S7: C-Terminus of four selected ORF6 homologs

Four examples of the C-terminus of ORF6 homologs, composed of multiple arrays of antiparallel beta sheets facing each other. Prediction was done using AlphaFold, color indicates pLDDT values.

Supplementary Tables

Table S1 - Accessions of proteins used for the initial search of homologs, minimum score and alignment length for the searches for each ORF

Table S2 - Foldseek results of ORF8, 9, 17, 21, 23 and 24.

Table S3 - apHPV overview, overview of all complete and incomplete discovered apHPVs, including: accessions, taxonomy, GC content, lengths, core-region position, circularity, defense systems.
